# Culture-based analysis of oral dysbiosis in post-oncological patients rehabilitated with obturator prostheses

**DOI:** 10.1080/20002297.2026.2698177

**Published:** 2026-08-04

**Authors:** Adam Bęben, Katarzyna Juszczuk, Ewa Kwapisz, Oliwia Chojnowska, Iwona Ordyniec-Kwaśnica, Katarzyna Garbacz

**Affiliations:** a Department of Dental Prosthetics, Medical Faculty, Medical University of Gdansk, Gdansk, Poland; b Department of Oral Microbiology, Medical Faculty, Medical University of Gdansk, Gdansk, Poland; c Wrzaskowscy Private Dentistry, Poland

**Keywords:** Maxillectomy, oral squamous cell carcinoma, obturator prosthesis, oral dysbiosis, post-oncological patients, culture-based, antimicrobial resistance

## Abstract

**Background:**

Post-oncological maxillary defects are commonly rehabilitated with obturator prostheses. This study compared the oral microbiota of post-oncological patients using obturators with that of control subjects wearing conventional removable dentures, focusing on clinically relevant, cultivable microorganisms.

**Methods:**

Post-oncological obturator users and denture-wearing controls were examined. Swabs from the oral mucosa, prosthesis surfaces, and resection sites were analyzed using culture-based microbiological methods, including species identification and antimicrobial susceptibility testing. Microbial, demographic, and clinical data were statistically analyzed.

**Results:**

Obturator users harbored a more pathogenic and antimicrobial-resistant oral microbiota than controls, with increased colonization by opportunistic pathogens such as *Staphylococcus aureus* (including MRSA), Gram-negative rods (*Klebsiella pneumoniae*), and yeast-like fungi. *S. aureus* remained significantly more prevalent in obturator users regardless of radiotherapy status. *Candida* spp. were prevalent in both groups, with *Candida albicans* predominating and *Candida tropicalis* more frequent among obturator users.

**Conclusions:**

Culture-based analysis demonstrated increased colonization with antimicrobial-resistant opportunistic pathogens in post-oncological patients using obturator prostheses, highlighting the need for intensified hygiene and preventive care.

## Introduction

Resection of the maxilla or mandible is a radical treatment method used for selected pathological lesions, such as extensive cysts or odontogenic tumours. Benign lesions that most often require resection include ameloblastomas and recurrent odontogenic cysts (keratocystic odontogenic cysts), both of which are characterised by aggressive growth and a high recurrence rate [1].

Squamous cell carcinoma (OSCC) is the most common malignant tumour of the oral cavity, accounting for approximately 90% of cases. The standard treatment involves surgical excision with a margin of healthy tissue. The resulting defect after resection of the jaw leads to pathological communication between the oral and nasal cavities, impairing basic functions such as speech and swallowing, as well as facial aesthetics [2,3].

There are two primary methods used to close such a communication: surgical reconstruction with autogenous tissues (flaps) or prosthetic treatment in the form of a custom–made obturator denture [4]. The obturator is currently one of the most commonly used methods of rehabilitation for patients after jaw resection [5,6]. Closing the defect with an obturator recreates the barrier between the nasal and oral cavities, allowing proper swallowing and articulation again, and also provides support for facial tissues (e.g. the floor of the nasal cavity and the infraorbital region), thereby preventing tissue collapse and cosmetic deformities [7]. It should be emphasised that extensive resections within the jaws cause significant aesthetic and functional problems and negatively affect the mental well-being and social integration of patients [8]. Postoperative defects in the jaw are often associated with speech impairment (hypernasality), difficulty in eating, and changes in facial features due to the collapse of the upper lip and cheek on the side of the resection. As a result, these patients may experience a reduced quality of life and require comprehensive prosthetic and speech therapy rehabilitation.

In clinical practice, immediate surgical reconstruction is not always feasible due to contraindications such as comorbidities, poor general condition, or scheduled radiotherapy, which increases the risk of flap healing complications. In such situations, prosthetic treatment is preferred. Obturators are also used in cases where observation and access to the tumour site are necessary—such a prosthesis provides temporary closure of the defect and can be easily removed during oncological check-ups, allowing for assessment of healing and possible recurrence of the disease. The use of an obturator prosthesis, therefore, has functional and oncological benefits [6,9]. Our University Dental Centre is one of the few in the country that offers rehabilitation with obturator prostheses to patients after oncological maxillary resection.

The role of oral microflora in the overall health of cancer patients remains frequently underestimated, despite growing evidence of its clinical relevance. Cancer treatment (radiotherapy, chemotherapy) can lead to changes in the oral environment, reduced saliva secretion, damage to the mucous membranes, and microbial imbalance [10]. The consequence is increased susceptibility to opportunistic infections, such as candidiasis or bacterial infections caused by pathogens typical for hospitalised patients. Recent reports suggest that oral microbiota may play a key role in the pathogenesis of many systemic diseases and influence their treatment [11,12]. However, microbiological data on clinically relevant oral microbiota changes in patients rehabilitated with obturator prostheses after oncological maxillectomy are limited.

The study aimed to compare the oral microbiota of post-oncological patients rehabilitated with obturator prostheses and control subjects with conventional removable dentures, using culture-based microbiological methods. In addition, selected demographic and clinical parameters of the study participants were analysed using a questionnaire to explore factors potentially associated with microbiological findings.

## Materials and methods

### Participants and sample collection

A total of 60 adult participants from the University Dental Centre of the Medical University of Gdańsk (Poland) were included in the study. The study group consisted of 30 obturator prosthesis wearers (18 women, 12 men) after partial resection of the jaw due to malignant tumour (squamous cell carcinoma). All patients in the study group were rehabilitated prosthetically at the University Dental Centre with a removable upper denture with an obturator restoring the excised tissues. Most of them underwent additional radiotherapy and/or postoperative chemotherapy. The control group consisted of 30 patients (19 women, 11 men) who used standard upper dentures (without an obturator), did not have oral cancer, and had not undergone chemotherapy and radiotherapy. Participants in both groups completed a standardised questionnaire that collected demographic data, general health information, and lifestyle habits. The questions also concerned the occurrence of oral diseases (caries, gingivitis, periodontitis), subjective oral complaints (dry mouth, burning sensation, difficulty swallowing, pain, inflammation of the corners of the mouth), as well as a history of smoking, alcohol consumption, and use of sedatives/sleeping pills. In addition, the duration of denture use, the method of use (whether it is removed at night, cleaned and stored dry or wet) and any repairs to the denture were recorded. The condition of the oral mucosa and any pathological changes were clinically assessed during the visit. From each study participant, oral samples from the following sites: the surface of the upper denture in use, oral vestibule (mucosa of the upper cheek on the denture side) and additionally in patients from the study group, also the area around the edges of the resection wound (i.e. the mucosa around the oro-nasal junction) were collected.

Prosthetic rehabilitation with obturator dentures was initiated 1–2 months after completion of oncological treatment, once the postoperative tissues were fully healed, non-bleeding, and non-tender. The duration of prosthetic treatment ranged from 3 to 4 weeks. Microbiological sampling was performed during routine follow-up visits after surgical treatment. Swabs were collected from the obturator dentures at various time points following completion of prosthetic treatment, and the duration of denture use at the time of sampling varied among patients, ranging from several months to two years. Recent antimicrobial therapy was an exclusion criterion; none of the included patients had received systemic antibiotics or antifungals within the preceding two months. All experimental procedures were approved by the local Bioethics Committee of the Medical University of Gdańsk (No. NKBBN/25/2023). Written informed consent was obtained from all participants.

### Microbiological study

A total of 150 oral samples were collected using sterile swabs on Stuart transport medium (Medlab-Products, Raszyn, Poland) and directly transported to the laboratory of the Department of Oral Microbiology at the Medical University of Gdansk. All swabs were subcultured on Columbia blood agar (GrasoBiotech, Starogard Gd., Poland), Chapman agar, MacConkey agar, D-Coccosel agar, and Sabouraud agar (bioMerieux, Marcy l’Etoile, France) and incubated aerobically at 37 °C for 18 to 24 h. Microbial strains were identified using a standard microbiological diagnostic procedure, including the API system (bioMérieux, Marcy-l’Étoile, France) and Candida-screen test [13]. Identification of microbial species was verified by the matrix-assisted laser desorption ionisation time-of-flight mass spectrometry (MALDI-TOF MS) method (Bruker Daltonics, MA, USA).

### Antimicrobial susceptibility

The antimicrobial susceptibility of non-duplicate staphylococcal strains was determined on Mueller-Hinton agar plates (Becton Dickinson, Franklin Lakes, NJ, USA) by the disk diffusion method and interpreted according to the EUCAST [14]. The following antimicrobial agents were tested: cefoxitin, gentamicin, erythromycin, clindamycin, tetracycline, ciprofloxacin, amoxicillin/clavulanic acid (Bio-Rad, Marnes la Coquette, France), and penicillin G (Oxoid, Basingstoke, UK). The inducible resistance to macrolide-lincosamide-streptogramin B (MLSB) was detected by the disk diffusion method with clindamycin (2 g) and erythromycin (15 g) discs positioned 15–26 mm apart [14]. MIC for vancomycin was determined by E-tests, in line with the manufacturer's instructions (AB Biodisc, Solna, Sweden).

Methicillin resistance was initially identified using cefoxitin (30 µg) and confirmed by the detection of PBP2a protein (OXOID™ PBP2' Latex Agglutination Test Kit, Basingstoke, England), and verified by the detection of the *mec*A gene according to Khairalla et al. and *mec*C according to Stegger et al. [15,16]. *S. aureus* ATCC®25923 (methicillin-susceptible) and *S. aureus* ATCC®43300 (MDR) were used as the reference strains.

The antimicrobial susceptibility of the non-duplicate strains of Gram-negative rods was determined using the disk diffusion method, as per the guidelines of the European Committee on Antimicrobial Susceptibility Testing (EUCAST) [14]. In total, the used antimicrobial agents included amikacin (30 µg), aztreonam (30 µg), cefepime (30 µg), ceftazidime (10 µg), ciprofloxacin (5 µg), gentamycin (10 µg), levofloxacin (5 µg), meropenem (10 µg), piperacillin/tazobactam (30µg/6µg), ticarcillin/clavulanic acid (75µg/10µg), trimethoprim/sulfamethoxazole (1.25µg/23.75 µg) (Oxoid, Basingstoke, England). The extended-spectrum *β*-lactamase (ESBL) was detected with discs with ceftazidime (30 µg), cefotaxime, and aztreonam (30 µg) placed at a distance of 20 mm from the centre of the disc with amoxicillin/clavulanic acid (20 µg/10 µg) [14].

### Statistical analysis

Statistical analysis for the purposes of the study was performed using Statistica 13.1 Pl. A significance level of *α* = 0.05 was adopted. Therefore, the results of the analyses were considered significant at *p* < 0.05. For *p* < 0.10, a statistical trend was determined. Due to the fact that the vast majority of dependent variables were described on a qualitative scale, Pearson's chi-square test of independence was most often used to verify the relationship between them and the independent variables. The strength of the relationship was evaluated using the *φ* (phi) coefficient. Comparisons of quantitative variables were performed using the Student's t-test (for age) or the Mann-Whitney U test (for prosthesis use time). When selecting the test, the normality of the dependent variable distributions in the compared groups (Shapiro-Wilk test) and the homogeneity of variance (Levene's test) were verified.

## Results

### Population study

The distribution of gender and age was comparable across both groups; women accounted for 60% of participants in both the study and control groups, and the average age was approximately 70 years. In the study group, the average age was 68.60 ± 11.98 years, and in the control group, it was 70.80 ± 11.20 years. Similarly, no significant differences were observed in residual dentition. In the control group, half of the patients were edentulous, while the other half retained some natural teeth (50%). In the study group, edentulism was slightly less prevalent, with most patients retaining several natural teeth (56.7%); the difference was not statistically significant. The prevalence of dental and periodontal conditions (including caries, gingivitis, and periodontitis) was also similar in both groups (*p* > 0.05). Caries was the most commonly reported issue (50%), particularly affecting residual teeth under future prostheses (Table 1).

**Table 1.** Oral health in participants from the control and study groups.

Oral health	Group		Statistics		
	Control (*n* = 30)	Study (*n* = 30)	*chi2*	*df*	*p*
Residual dentition	15 (50.0%)	17 (56.7%)	0.27	1	0.605
Caries	15 (50.0%)	14 (46.7%)	0.07	1	0.796
Gingivitis	8 (26.7%)	9 (30.0%)	0.08	1	0.775
Periodontitis	9 (30.0%)	8 (26.7%)	0.08	1	0.775
Dry mouth	4 (13.3%)	15 (50.0%)	9.32	1	0.002
Burning mouth	2 (6.7%)	7 (23.3%)	3.27	1	0.071
Difficulty swallowing	1 (3.3%)	4 (13.3%)	1.96	1	0.161
Angular cheilitis	0 (0.0%)	7 (23.3%)	7.92	1	0.005

Note: The table shows the numbers and percentages of people experiencing symptoms in each group. *Chi2* – Pearson's chi-square test value; *df* – degrees of freedom; *p* – statistical significance level.

Subjective oral complaints showed statistically significant differences. A higher proportion of patients in the obturator group reported symptoms such as dryness, burning sensation, swallowing difficulties, or angular cheilitis (76.7% vs. 33.3%; *p* = 0.001). The most pronounced difference was observed in xerostomia, which was reported by 50% of patients in the study group compared to 13.3% in the control group (*p* = 0.002). Angular cheilitis was exclusively observed in the obturator group (23.3%; *p* 0.005). Burning sensation and swallowing difficulties were also more frequent among obturator users, but this was not statistically significant (*p* > 0.05) (Table 1).

The average self-reported duration of current denture use was 14 months in the study group and 11 months in the control group, with no significant difference between the groups. The majority of patients (~80% in both groups) routinely removed their dentures at night and stored them in a dry environment. A non-significant trend was noted for dry storage being more common among control group patients (88%) compared to obturator users (66.7%) (*p* > 0.05).

As expected, the study group demonstrated significant differences in general health characteristics due to prior oncological treatment. Patients in this group were more likely to have been hospitalised in the past two years (53.3% vs. 26.7%; *p* < 0.05), and had significantly higher rates of chemotherapy 23.3% vs. 0%; *p* = 0.005) and head and neck radiotherapy (53.3% vs. 0%; *p* < 0.001) (Table 2).

**Table 2.** Health status of participants from the control and study groups.

Health status	Group		Statistics		
	Control (*n* = 30)	Study (*n* = 30)	*chi2*	*df*	*p*
Feeling healthy	22 (73.3%)	25 (83.3%)	0.88	1	0.347
Hospitalisation in the last two years	8 (26.7%)	16 (53.3%)	4.44	1	0.035
Chemotherapy	0 (0.0%)	7 (23.3%)	7.92	1	0.005
Radiotherapy	0 (0.0%)	16 (53.3%)	21.82	1	<0.001
Get treatment now	23 (76.7%)	24 (80.0%)	0.10	1	0.754

Note: The table shows the numbers and percentages of “yes” responses in each group. *Chi2 –* Pearson's chi-square test value; *df* – degrees of freedom; *p* – statistical significance level.

No statistically significant differences were observed in the prevalence of other chronic (e.g. cardiovascular disease, diabetes, thyroid disorders, osteoporosis) and infectious diseases (hepatitis, AIDS, tuberculosis), which were similarly distributed across both groups. However, nervous system disorders were more common in the control group (23% vs. 3%; *p* < 0.05), though most were mild (Table 3).

**Table 3.** Prevalence of chronic and infectious diseases in participants from the control and study groups.

Disease	Group		Statistics		
	Control (*n* = 30)	Study (*n* = 30)	*chi2*	*df*	*p*
Cancer, neoplasm	3 (10.0%)	28 (93.3%)	41.71	1	<0.001
Heart disease	12 (40.0%)	10 (33.3%)	0.29	1	0.592
Other cardiovascular diseases	16 (53.3%)	15 (50.0%)	0.07	1	0.796
Blood vessel diseases	10 (33.3%)	10 (33.3%)	0.00	1	1.000
Lung diseases	2 (6.7%)	2 (6.7%)	0.00	1	1.000
Digestive system diseases	7 (23.3%)	4 (13.3%)	1.00	1	0.317
Liver diseases	3 (10.0%)	2 (6.7%)	0.22	1	0.640
Urinary tract diseases	6 (20.0%)	5 (16.7%)	0.11	1	0.739
Metabolic disorders	4 (13.3%)	6 (20.0%)	0.48	1	0.488
Thyroid diseases	10 (33.3%)	14 (46.7%)	1.11	1	0.292
Nervous system diseases	7 (23.3%)	1 (3.3%)	5.19	1	0.023
Bone and joint diseases	13 (43.3%)	17 (56.7%)	1.07	1	0.302
Blood and coagulation diseases	3 (10.0%)	6 (20.0%)	1.18	1	0.278
Rheumatic disease	7 (23.3%)	2 (6.7%)	3.27	1	0.071
Osteoporosis	1 (3.3%)	5 (16.7%)	2.96	1	0.085
Infectious diseases	2 (6.7%)	1 (3.3%)	0.35	1	0.554
Hepatitis A	0 (0.0%)	1 (3.3%)	1.02	1	0.313
Hepatitis B	1 (3.3%)	0 (0.0%)	1.02	1	0.313
Hepatitis C	0 (0.0%)	0 (0.0%)	–	–	–
AIDS	0 (0.0%)	0 (0.0%)	–	–	–
Tuberculosis	0 (0.0%)	0 (0.0%)	–	–	–
Venereal diseases	0 (0.0%)	0 (0.0%)	–	–	–

Note: The table shows the numbers and percentages of patients in each group. *Chi2* – Pearson's chi-square test value; *df* – degrees of freedom; *p* – statistical significance level.

Smoking and alcohol use were comparable; 33.3% of participants in the control group and 23.3% with obturators were smokers. Slightly more participants in the study group (33.3%) than in the control group (16.7%) consumed alcohol regularly. Notably, patients in the obturator group were significantly more likely to report chronic use of sleeping pills or sedatives (30% vs. 7%; *p* = 0.038), potentially indicating greater sleep disturbances or psychological stress.

### Isolation and identification of microorganisms

Patients using obturator prostheses were significantly more likely to exhibit disturbed oral microbiota compared to those using conventional dentures, in whom typical microflora was far more prevalent (96.7% vs. 63.3%; *p* = 0.001). The normal microflora mainly consisted of *viridans* group streptococci, *Neisseria* spp., and *Corynebacterium* spp.

Microbiological analysis revealed significant differences in colonisation patterns between the groups. Staphylococci were isolated from 86.7% of patients in the obturator group compared to only 33.3% in the control group (*p* < 0.001) (Table 4). The most commonly isolated species was *Staphylococcus aureus*, found in 80% of patients in the study group and 20% of the controls (*p* < 0.001). Coagulase-negative staphylococci, including *S. saprophyticus*, *S. epidermidis*, and *S. lugdunensis*, were rare and distributed evenly across both groups (*p* > 0.05, not statistically significant) (Table 5).

**Table 4.** Prevalence of microbial groups in participants from the control and study groups.

Microbial group	Group		Statistics		
	Control (*n* = 30)	Study (*n* = 30)	*chi2*	*df*	*p*
Staphylococci	10 (33.3%)	26 (86.7%)	17.78	1	<0.001
Gram-negative rods	9 (30.0%)	19 (63.3%)	6.70	1	0.010
Yeast-like fungi	21 (70.0%)	29 (96.7%)	7.68	1	0.006

Note: The table shows the numbers and percentages of “yes” responses in each group. *Chi2* – Pearson's chi-square test value; *df* – degrees of freedom; *p* – statistical significance level.

**Table 5.** Prevalence of microbial species in participants from the control and study groups.

Microbial species	Group		Statistics		
	Control (*n* = 30)	Study (*n* = 30)	*chi2*	*df*	*p*
Staphylococci					
*Staphylococcus aureus*	6 (20.0%)	24 (80.0%)	21.60	1	<0.001
*Staphylococcus saprophyticus*	3 (10.0%)	1 (3.3%)	1.07	1	0.301
*Staphylococcus epidermidis*	1 (3.3%)	1 (3.3%)	0.00	1	1.000
*Staphylococcus lugdunensis*	1 (3.3%)	0 (0.0%)	1.02	1	0.313
Gram-negative rods					
*Citrobacter freundii*	3 (10.0%)	0 (0.0%)	3.16	1	0.076
*Eschericha coli*	2 (6.7%)	4 (13.3%)	0.74	1	0.389
*Klebsiella oxytoca*	2 (6.7%)	4 (13.3%)	0.74	1	0.389
*Klebsiella pneumoniae*	1 (3.3%)	6 (20.0%)	4.04	1	0.044
*Enterobacter aerogenes*	1 (3.3%)	3 (10.0%)	1.07	1	0.301
*Enterobacter cloacae*	1 (3.3%)	3 (10.0%)	1.07	1	0.301
*Morganella morganii*	0 (0.0%)	1 (3.3%)	1.02	1	0.313
*Serratia odorifera*	1 (3.3%)	0 (0.0%)	1.02	1	0.313
*Serratia marcensens*	0 (0.0%)	1 (3.3%)	1.02	1	0.313
*Pseudomonas aeruginosa*	3 (10.0%)	3 (10.0%)	0.00	1	1.000
Yeast-like fungi					
*Candida albicans*	10 (33.3%)	14 (46.7%)	1.11	1	0.292
*Candida tropicalis*	7 (23.3%)	14 (46.7%)	3.59	1	0.058
*Candida glabrata*	3 (10.0%)	6 (20.0%)	1.18	1	0.278
*Candida guilliermondii*	2 (6.7%)	1 (3.3%)	0.35	1	0.554
*Candida kefyr*	2 (6.7%)	1 (3.3%)	0.35	1	0.554
*Candida krusei*	2 (6.7%)	5 (16.7%)	1.46	1	0.228
*Candida lipolytica*	2 (6.7%)	1 (3.3%)	0.35	1	0.554
*Candida dubliniensis*	1 (3.3%)	1 (3.3%)	0.00	1	1.000
*Candida lusitaniae*	1 (3.3%)	3 (10.0%)	1.07	1	0.301
*Saccharomyces cerevisiae*	4 (13.3%)	4 (13.3%)	0.00	1	1.000
*Rhodotorula mucilaginosa*	0 (0.0%)	1 (3.3%)	1.02	1	0.313

The table shows the numbers and percentages of strains in each group. *Chi2* – Pearson's chi-square test value; *df* – degrees of freedom; *p* – statistical significance level.

Gram-negative rods (*Enterobacterales* and non-fermenting rods) were more frequently isolated from obturator users (63.3%) than controls (30.0%) (*p* < 0.05) (Table 4). In particular, *Klebsiella pneumoniae* was found significantly more often in the study group (20% vs. 3.3%; *p* = 0.044). Other enterobacteria such as *Citrobacter freundii*, *Escherichia coli*, *Klebsiella oxytoca*, *Enterobacter* spp., *Morganella* spp., and *Serratia* spp. appeared sporadically without significant differences (*p* > 0.05). *Citrobacter freundii* was exclusively isolated from three individuals in the control group. *Pseudomonas* spp. (non-fermenting rod) occurred with equal frequency in both groups (10% each) (Table 5).

Yeast-like fungi (*Candida* spp.) were more prevalent in the obturator group (96.7%) than in the control group (70.0%; *p* > 0.05), indicating a greater tendency toward fungal colonisation (Table 4). *Candida albicans* was the most frequently isolated species in both groups (46.7% vs. 33.3%; *p* > 0.05), though nearly all patients who had undergone radiotherapy (i.e. the study group) tested positive for *Candida* spp., including non-*albicans* strains. *Candida tropicalis*, a common non-*albicans* species, was twice as frequent in the study group (46.7%) as in the control group (23.3%), with the difference approaching significance (*p* = 0.058). Other non-*albicans* species were also more common in the study group, though found infrequently: *C. glabrata* (20% vs. 10%), *C. krusei* (16.7% vs. 6.7%), *C. lusitaniae* (10% vs. 3%), *C. guilliermondii*, and *C. kefyr*. *Rhodotorula mucilaginosa* and *Saccharomyces cerevisiae* were isolated from four patients in both groups, likely related to probiotic use (Table 5).

### Antimicrobial susceptibility

**Figure 1.**
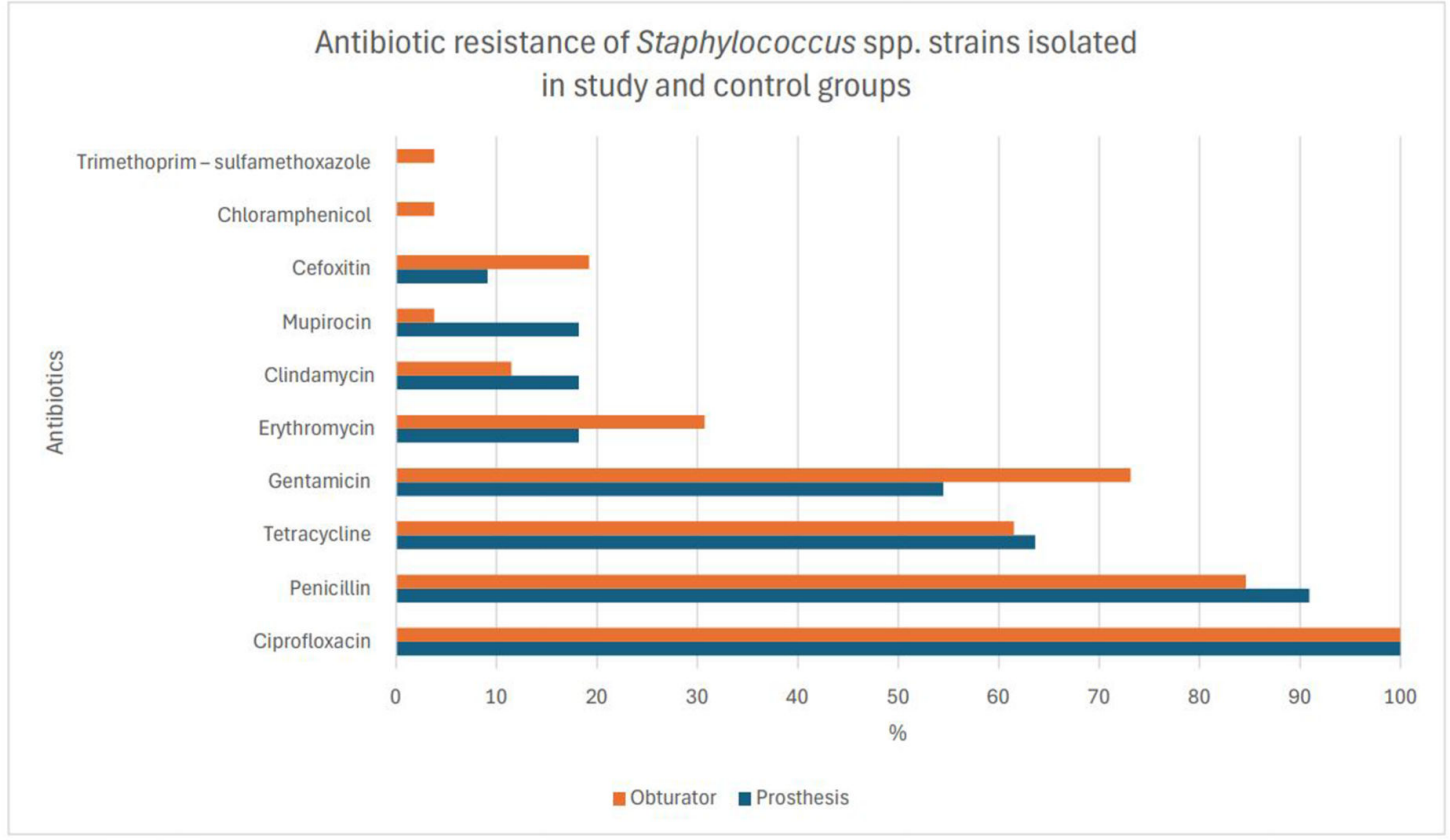
Antibiotic resistance of *Staphylococcus* spp. strains isolated in study and control groups.

According to the EUCAST recommendation, all isolated *S. aureus* strains were resistant to ciprofloxacin (100%), followed by resistance to penicillin (86.5%), tetracycline (62.2%), gentamycin (67.6%), erythromycin (27.0%), cefoxitin (16.2%), clindamycin (13.5%), mupirocin (8.1%), chloramphenicol (2.7%), and trimethoprim-sulfamethoxazole (2.7%). The differences in antibiotic resistance between strains isolated in the study and control groups were not significant (*p* > 0.05) (Figure 1). Four methicillin-resistant *S. aureus* (MRSA) strains (10.8%) were isolated, three from patients with obturators and one in the control group. Methicillin resistance was observed in two coagulase-negative staphylococcal strains, *S. epidermidis* and *S. saprophyticus,* both isolated from patients with obturators. All methicillin-resistant staphylococcal species carried *mec*A gene.

**Figure 2.**
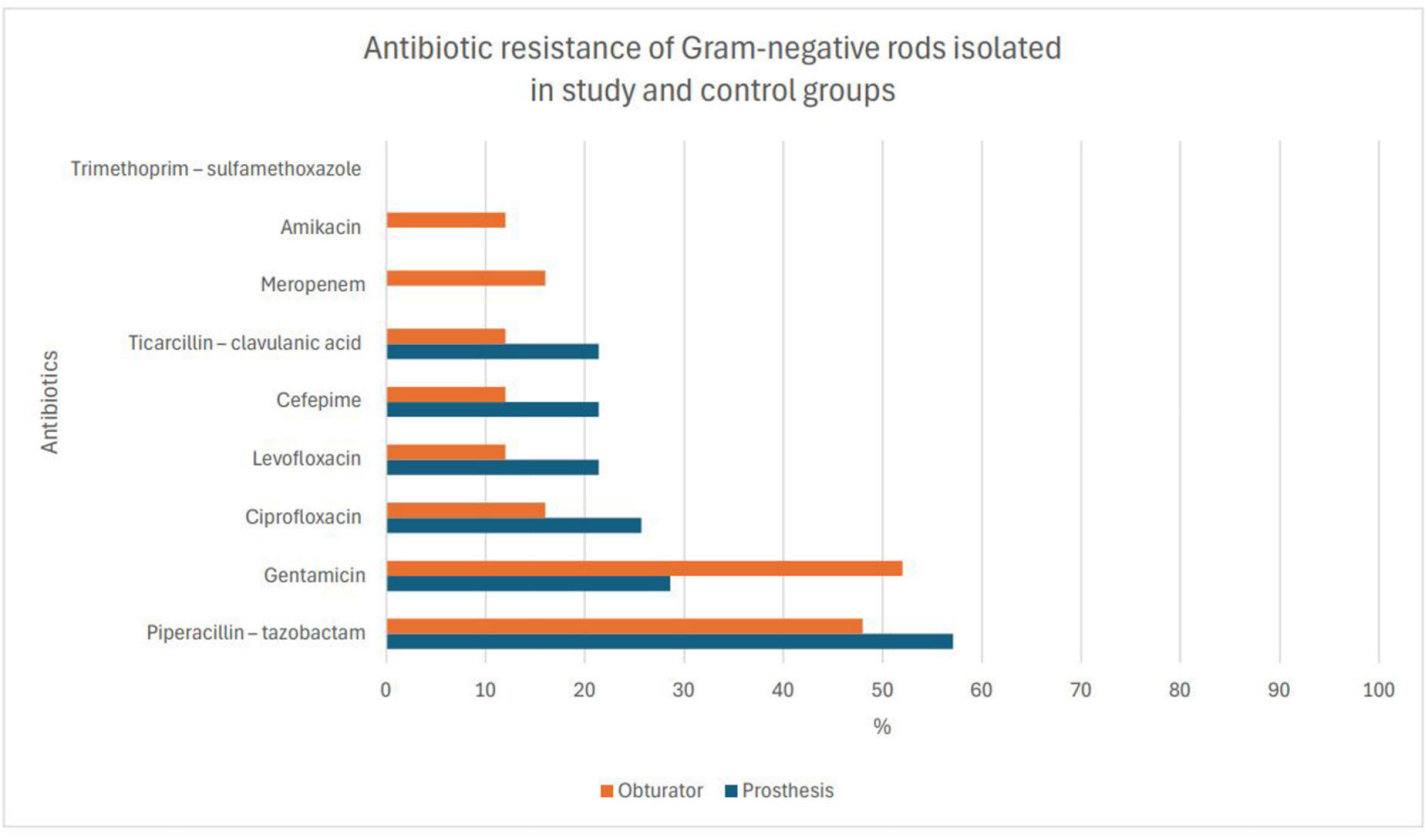
Antibiotic resistance of Gram-negative rods isolated in study and control groups.

In total, Gram-negative rods (GNR) isolated in study and control group were resistant to piperacillin/tazobactam (51.3%), gentamycin (43.6%), ciprofloxacin (23.1%), levofloxacin (15.4%), cefepime (15.4%), ticarcillin/clavulanic acid (15.4%), meropenem (11.1%), and amikacin (7.7%). All strains were susceptible to co-trimoxazole (trimethoprim/sulfamethoxazole). No strain produced extended-spectrum *β*-lactamase. The differences in antibiotic resistance between strains isolated in the study and control groups were not significant (*p* > 0.05) (Figure 2).

### Stratified analysis of radiotherapy as a potential confounder

To address the potential confounding effects of oncological treatment, stratified analyses were performed within the obturator group, comparing patients with and without prior radiotherapy. Radiotherapy was associated only with dysphagia (*φ* = 0.37) and was not linked to significant differences in other clinical symptoms, predominant oral microflora, or antimicrobial resistance patterns. Importantly, the main microbiological findings persisted after exclusion of radiotherapy-treated patients, including increased prevalence of staphylococci (*φ* = 0.54), Gram-negative rods (*φ* = 0.33), and yeast-like fungi (*φ* = 0.36). When patients with a history of radiotherapy were excluded, *S. aureus* remained significantly more prevalent in the obturator group compared with controls (71.4% vs. 20.0%; χ^2^ = 10.91, *p* = 0.001), with a large effect size (*φ* = 0.50). Comparable results were observed when patients with prior radiotherapy were included (80.0% vs. 20.0%; χ^2^ = 21.60, *p* < 0.001), again demonstrating a strong association (*φ* = 0.60) (Tables 6 and 6A).

**Table 6.** Comparison of oral symptoms and predominant microorganisms in obturator patients with and without prior radiotherapy.

	Study group		Statistics		
	With radiotherapy (*n* = 16)	Without radiotherapy (*n* = 14)	*chi2*	*df*	*p*
Dry mouth	10 (62.5%)	5 (35.71%)	2.14	1	0.143
Burning mouth	3 (18.8%)	4 (28.6%)	0.40	1	0.526
Difficulty swallowing	4 (25.0%)	0 (0.0%)	4.04	1	0.044
Angular cheilitis	3 (18.8%)	4 (28.6%)	0.40	1	0.526
Staphylococci	15 (93.8%)	11 (78.6%)	1.49	1	0.222
*S. aureus*	14 (87.5%)	10 (71.4%)	1.21	1	0.272
Gram-negative rods	11 (68.8%)	8 (57.1%)	0.43	1	0.510
Yeast-like fungi	16 (100.0%)	13 (92.9%)	1.18	1	0.277
MRSA	3 (18.8%)	0 (0.0%)	2.92	1	0.088
MSSA	11 (68.8%)	10 (71.4%)	0.03	1	0.873

Note: The table shows the numbers and percentages of “yes” responses in each group. *Chi2 –* Pearson's chi-square test value; *df* – degrees of freedom; *p* – statistical significance level, MRSA – methicillin-resistant *S. aureus*, MSSA – methicillin-susceptible *S. aureus*.

**Table 6A.** Comparison of oral symptoms and predominant microorganisms between control subjects and obturator patients without prior radiotherapy.

	Group		Statistics		
	Control (*n* = 30)	Study group without radiotherapy (*n* = 14)	*chi2*	*df*	*p*
Dry mouth	4 (13.3%)	5 (35.7%)	2.94	1	0.086
Burning mouth	2 (6.7%)	4 (28.6%)	3.89	1	0.049
Difficulty swallowing	1 (3.3%)	0 (0.0%)	0.48	1	0.490
Angular cheilitis	0 (0.0%)	4 (28.6%)	9.43	1	0.002
Staphylococci	10 (33.3%)	11 (78.6%)	7.83	1	0.005
*S. aureus*	6 (20.0%)	10 (71.4%)	10.91	1	0.001
Gram-negative rods	9 (30.0%)	8 (57.1%)	2.97	1	0.085
Yeast-like fungi	21 (70.0%)	13 (92.9%)	2.84	1	0.092
MRSA	1 (3.3%)	0 (0.0%)	0.48	1	0.490
MSSA	5 (16.7%)	10 (71.4%)	12.74	1	<0.001

Note: The table shows the numbers and percentages of “yes” responses in each group. *Chi2 –* Pearson's chi-square test value; *df* – degrees of freedom; *p* – statistical significance level, MRSA – methicillin-resistant *S. aureus*, MSSA – methicillin-susceptible *S. aureus*.

## Discussion

The microbial community of the mouth coexists harmoniously with the host, and this symbiosis benefits both. Exogenous, commonly pathogenic bacteria can colonise the mouth following loss or disruption of the resident microbiota, thereby predisposing individuals to various disorders [11,12].

The present study revealed that individuals using obturators were significantly more likely to exhibit disturbed oral microbiota than wearers of conventional dentures, who more frequently demonstrated typical microflora. Numerous studies have shown that commensal bacteria in the oral cavity protect against colonisation by pathogenic microorganisms [12,17].

The oral microbiota of cancer patients with obturators exhibited signs of dysbiosis, including potentially pathogenic antimicrobial-resistant species such as *Staphylococcus aureus*, *Klebsiella pneumoniae*, and *Candida* spp. This pattern can be attributed to several contributing factors, most notably prior radiotherapy and chemotherapy. Head and neck radiotherapy, standard in patients treated for jaw tumours, causes damage to the salivary glands and leads to persistent xerostomia. Reduced salivary flow compromises the oral cavity’s natural defence mechanisms. Saliva has cleansing, lubricating, and antimicrobial functions; its deficiency promotes the colonisation of mucosal surfaces by pathogens. In our results, the percentage of patients reporting dry mouth was significantly higher in the obturator group (50%) than in the control group (13%), consistent with a history of radiotherapy. Xerostomia is a significant risk factor for the development of bacterial infections as well as oral candidiasis [6,17,18].

Colonisation by *S. aureus* was found in 70% of oncology patients using obturators, compared to only 17% in the control group. *S. aureus* is not a component of the typical oral flora; its presence is often associated with nasal or skin colonisation in hospitalised individuals and may reflect healthcare-associated exposure. In our patient group, several factors likely contributed to the increased colonisation by *S. aureus*, including mucosal damage caused by radiotherapy, frequent hospitalisations, prior antibiotic therapy, and immunosuppression following cancer treatment [19,20]. The role of *S. aureus* in oral infections remains a subject of debate, as it is found in healthy carriers. Although the anterior nares are the main niche, 15–50% of colonised individuals are not nasal carriers. The oral cavity may be the primary or secondary site of colonisation, either transiently or permanently. Recent studies have shown that it is considered an important, often overlooked source of infection [21,22]. Oral carriage can lead to endogenous or cross-infections, with immunocompromised individuals after radiotherapy or chemotherapy being at greater risk. The presence of *S. aureus* in saliva increases the likelihood of aspiration pneumonia and is associated with severe infections, such as bacteremia and infective endocarditis. Colonised individuals are at 11.5 times greater risk of endogenous infections [23,24].

Methicillin-resistant *S. aureus* strains (MRSA), isolated in this study predominantly from patients using obturator prostheses, represent a significant clinical concern. Their resistance extends beyond *β*-lactam antibiotics to multiple commonly used antimicrobial classes, increasing the risk of treatment failure across a wide range of infections. MRSA can cause severe diseases, including pneumonia, endocarditis, and sepsis, and readily spreads in hospitals, outpatient healthcare settings, and the community. Asymptomatic carriage is common, facilitating unrecognised transmission, while individuals with compromised immune systems remain particularly susceptible to severe and potentially life-threatening infections. [25–27].

Sroussi et al. emphasised that patients undergoing head and neck cancer therapy are at elevated risk for opportunistic oral infections, both bacterial and fungal, due to local and systemic immune dysfunction [28]. Our findings are consistent with this observation: almost all patients in the obturator group had positive cultures for *Candida* spp., and over half were colonised with Gram-negative rods (GNR) such as *Klebsiella*, *Pseudomonas*, *Escherichia coli*, and *Enterobacter*. The persistence of GNR in the oral cavity makes patients, especially those who are hospitalised or immunocompromised, susceptible to bacterial infections such as pneumonia, bacteraemia and urinary tract infections [29]. Their presence in the oral cavity of immunocompromised individuals, particularly *K. pneumoniae*, raises concerns about the potential for secondary infections such as pneumonia. The threat associated with *K. pneumoniae* colonisation stems mainly from its widespread antibiotic resistance, including the production of extended-spectrum beta-lactamases (ESBLs) and carbapenemases [30]. Although we did not detect such resistance, the results emphasised the importance of enhanced dental control, hygiene instructions, and infection prevention strategies in oncology patients rehabilitated with obturator prostheses.

Many authors have noted that although obturators offer significant functional benefits, they can promote local colonisation by *Candida* spp. and bacteria if not properly maintained [18,31,32]. Our findings support this observation; *Candida* spp. were isolated from 96.7% of patients in the obturator group, compared with 70% in the conventional denture group. While colonisation in the control group was also relatively high, likely due to the porous nature of acrylic dentures and age-related susceptibility, the significantly higher prevalence in the study group points to additional oncological risk factors. Previous studies indicate that nearly all patients receiving head and neck radiotherapy experience some degree of oral candidiasis during treatment [18]. Lyons et al. demonstrated a sharp increase in *C. albicans* colonisation on obturator surfaces in irradiated patients, with antifungal treatment and relining offering only temporary relief [18]. Consistent with these findings, our study revealed that *C. albicans* was the predominant fungal species in the obturator group. *C. tropicalis* was also isolated more frequently in this group. This finding may have clinical significance, as *C. tropicalis* is a non-albicans *Candida* species associated with opportunistic, persistent, and recurrent infections, particularly in patients with underlying health conditions [33]. Its increased prevalence in patients wearing obturator dentures may reflect changes in the oral environment following cancer treatment, including postoperative anatomical changes, denture-related retention areas, and favourable conditions for microbial adhesion [18]. Furthermore, the potential virulence and drug resistance of *C. tropicalis* suggest that its presence in patients with obturators may not only result in colonisation but also cause oral candidiasis and recurrent denture-associated infections [33].

Notably, 23% of patients in this group exhibited clinical signs of angular cheilitis, a condition often associated with *Candida* overgrowth. This symptom was absent in the control group. Additionally, our results align with those of Oza et al., who reported a key role of *S. aureus* in the pathogenesis of angular cheilitis [34,35]. In our cohort, *S. aureus* colonisation was significantly more frequent in the obturator group, suggesting that the presence of both *S. aureus* and *Candida* spp. in these patients likely represented actual infection rather than mere colonisation.

The finding that *S. aureus*, including methicillin-resistant strains, was more prevalent among obturator users, although not directly associated with radiation exposure in stratified analyses, is consistent with previous reports. Systematic and prospective microbiome studies in head and neck cancer patients have shown that radiotherapy can indirectly alter the oral ecosystem through mechanisms such as salivary gland dysfunction, mucosal injury, and immune dysregulation [36,37]. Although the present culture-based approach does not capture community-level dynamics and therefore cannot be directly compared with sequencing-based studies, the predominance of Gram-positive organisms such as *S. aureus* aligns with reports describing a relative increase in Gram-positive taxa following radiotherapy [36,37]. Taken together, these findings suggest that radiotherapy may create permissive biological conditions (e.g. xerostomia and impaired host defences), whereas the observed enrichment of *S. aureus* among obturator users is more likely related to local anatomical changes and prosthesis-associated factors rather than to radiation exposure alone.

A key practical implication of our findings is the need for enhanced preventive care and patient awareness. The high rate of colonisation in the oncology group underscores the importance of routine dental check-ups, which should include mucosal assessments, professional denture cleaning, and, if necessary, prosthesis relining or replacement. Patient instruction should emphasise proper hygiene, including daily removal and thorough cleaning of the obturator using special brushes and antimicrobial agents. Pereira-Cenci and Lyons have demonstrated that the porous surface of acrylic prostheses facilitates *Candida* adherence and biofilm formation, particularly when worn continuously and inadequately cleaned [38,39]. As such, removing the prosthesis at night to allow mucosal rest is strongly recommended. Most patients in our study reported following this guideline, although a subset wore their obturators continuously.

This study has several limitations. Firstly, the anatomical characteristics of maxillary defects were not stratified, although microbiological outcomes may vary by communication type and defect size. In addition, functional and behavioural confounders such as oral food intake, chewing efficiency, salivary flow, denture wear time, and detailed prosthetic characteristics were not quantitatively assessed. Furthermore, the microbiological analysis relied on culture-dependent methods, which may underestimate overall microbial diversity and fail to capture non-culturable or low-abundance taxa, unlike culture-independent microbiome approaches. Nevertheless, the study provides a clinically feasible, standardised assessment of viable microorganisms and antimicrobial resistance patterns in a well-defined, rarely described patient population, offering valuable baseline data for future longitudinal and microbiome-based studies.

In summary, patients rehabilitated with obturator prostheses following oncologic maxillary resection exhibit clear signs of oral dysbiosis, characterised by frequent colonisation with opportunistic antimicrobial-resistant pathogens, including *Staphylococcus aureus*, *Klebsiella pneumoniae*, and *Candida* species. This is likely a consequence of oncologic therapies, particularly radiotherapy and chemotherapy, which lead to xerostomia and impaired mucosal defences. Clinically, these patients report more frequent oral complaints, including dryness, burning, and angular cheilitis, and may be at increased risk of systemic infections. These findings underscore the importance of interdisciplinary care that extends beyond prosthetic rehabilitation, encompassing infection prevention, individualised monitoring, and patient education on prosthesis hygiene.
